# Psychosocial stress and autoimmune thyroid disease: a systematic review of clinical phenotypes and vulnerability factors

**DOI:** 10.1016/j.cpnec.2026.100382

**Published:** 2026-09-22

**Authors:** José Manuel Comprido, Ana Carolina D. Salina, Vitor Vasconcellos F. da Silva, Paulo Roberto Haidamus de O. Bastos, Renata Boschi Portella, Danielle Bogo, Marco Aurélio Vinhosa Bastos

**Affiliations:** a Federal University of Mato Grosso do Sul (UFMS), School of Medicine, Campo Grande, Brazil; b Anhanguera-Uniderp University, School of Medicine, Campo Grande, Brazil; c Graduate Program in Health and Development, Federal University of Mato Grosso do Sul (UFMS), Campo Grande, Brazil

**Keywords:** Psychological stress, Autoimmune disease, Thyroid, Hashimoto's thyroiditis, Graves' disease

## Abstract

Psychological stress has been proposed as a potential trigger or modulator of autoimmune thyroid diseases, but evidence remains fragmented. This systematic review examined the relationship between stress and thyroid autoimmunity, with particular attention to Hashimoto's thyroiditis and Graves' disease, highlighting the critical methodological distinction between stressor exposure (objective events) and the subjective stress response (psychological burden). PubMed, Embase, Scopus, and PsycINFO were searched for controlled human studies; 25 studies involving a total of N = 117,034 participants met the inclusion criteria and were synthesized narratively across seven analytical categories. Substantial heterogeneity precluded a meta-analysis, partly driven by the predominance of measures of stressful life events (15 studies; 60%) over assessments of the subjective stress response (4 studies; 16%). Findings demonstrate that associations are critically phenotype-dependent and contingent on the stress construct evaluated. For Graves' disease, evidence reveals a robust association with stressful life events, acting as a significant precipitating factor for disease onset and relapse. In contrast, Hashimoto's thyroiditis is more closely linked to a high subjective stress response, which functions as an amplifier of disease activity and autonomic dysfunction. Early-life adversity and genetic susceptibility (e.g., HLA-Cw7) emerge as key vulnerability factors that reset biological regulation thresholds through ‘biological embedding’. Mechanistically, stress contributes to neuroendocrine–immune dysregulation via hypothalamic-pituitary-adrenal axis and sympathetic activation, facilitating the breakdown of immunological tolerance. These findings highlight the clinical necessity of incorporating psychosocial assessments into the management of autoimmune thyroid conditions.

## Highlights

• Stress–AITD associations appear phenotype-dependent and heterogeneous.
• Evidence was strongest for Graves' disease onset, relapse, and course.
• Hashimoto's thyroiditis was mainly linked to psychological burden.
• Early-life adversity and genetic susceptibility may shape vulnerability.
• Stress may act as a precipitant or amplifier in susceptible individuals.

## Graphical abstract

**Figure:**
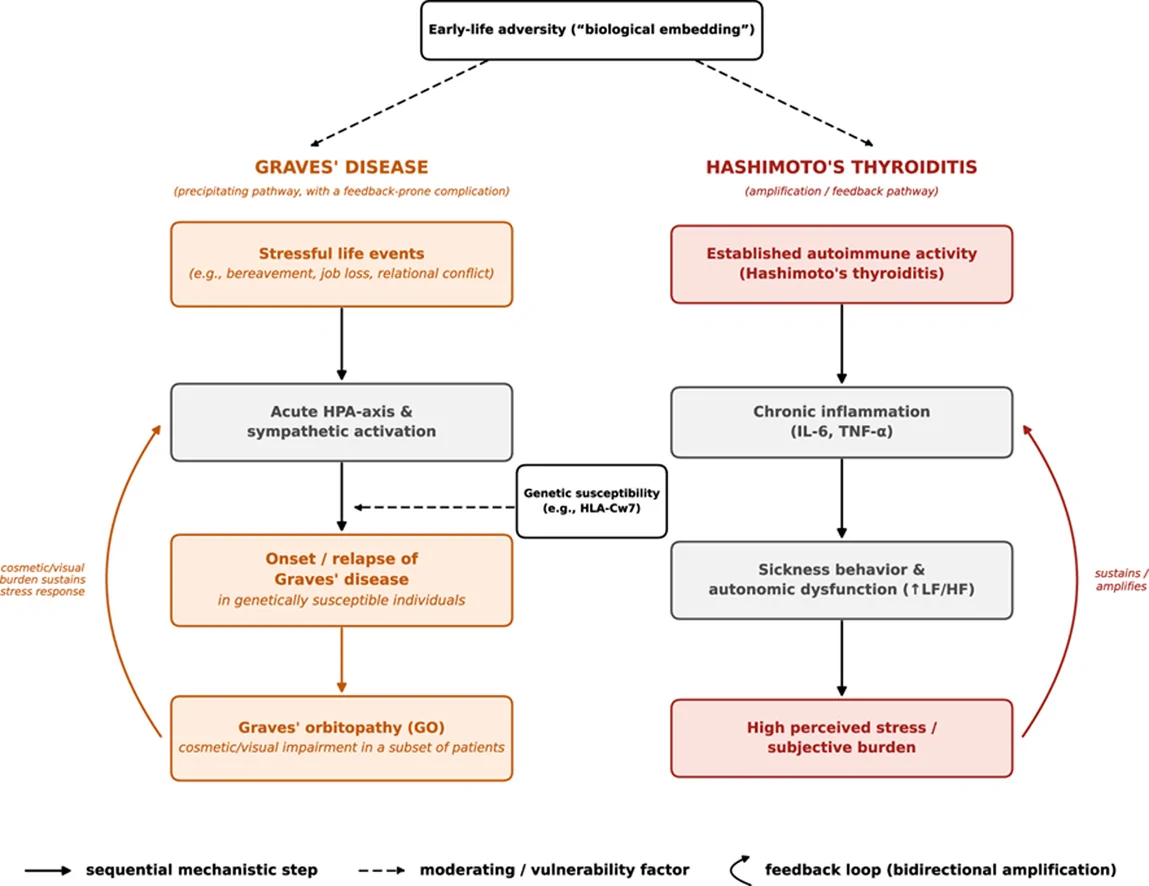

## 1 Introduction

Stress is commonly used as an umbrella concept to describe circumstances in which environmental demands surpass the individual's perceived psychological and physiological resources to manage them effectively [1,2]. While acute stress promotes short-term homeostasis, contemporary lifestyles characterized by cognitive overload, work intensification, and psychosocial demands have increased exposure to chronic psychological stress [3,4].

A critical distinction in this field is between stressor exposure—the objective environmental events—and the stress response, which encompasses the cognitive, emotional, and biological reactions these events evoke [2]. Empirical evidence suggests that the subjective psychological burden and the degree to which an individual perceives their circumstances as overwhelming are often stronger predictors of health decline than mere exposure to stressful events [2]. In modern contexts, the accumulation of daily hassles, such as persistent work overload and frequent interruptions, can build up over time into a state of allostatic load, the cumulative physiological toll of chronically taxed stress-response systems, disrupting typical psychological and physiological functioning [2,[5], [6], [7]]. This distinction may also be relevant to autoimmune thyroid diseases (AITD), in which stress-related biological responses interact with immune and endocrine regulatory pathways [[7], [8], [9]].

AITD, primarily comprising Graves' disease (GD) and Hashimoto's thyroiditis (HT), are T cell-mediated organ-specific disorders resulting from a dysregulation of the immune system and a subsequent loss of tolerance toward self-antigens [[8], [9], [10]]. While GD is characterized by thyrotoxicosis due to stimulating autoantibodies against the TSH receptor, HT leads to hypothyroidism through the destruction of thyroid parenchyma by autoreactive T cells and antibodies against thyroglobulin (Tg-Ab) and thyroid peroxidase (TPO-Ab) [10,11]. The pathogenesis of these conditions is multifactorial and arises from a complex interaction between genetic susceptibility (such as HLA-DR3 variants) and environmental triggers, including iodine intake, infections, and chronic psychosocial stress [8,9,11]. Chronic stress acts as a contributor to immune dysregulation by fostering a state of systemic low-grade inflammation [7,9].

The underlying regulatory mechanism of the thyroid is governed by a “secrete-and-grow” circuit, where thyroid-stimulating hormone (TSH) induces both the secretion of thyroid hormones (T3 and T4) and the proliferation of thyroid follicular cells. This system ensures homeostatic balance via a negative feedback loop [10]. In susceptible individuals, the hypothalamic-pituitary-adrenal (HPA) axis and the sympathetic nervous system (SNS) link stress exposure to pathophysiological processes [7,9]. Under prolonged stress, the HPA axis may lose its adaptive function, leading to impaired negative feedback and glucocorticoid receptor (GR) resistance [9]. This neuroendocrine imbalance results in diminished immunosuppressive effects, effectively shifting the immune environment from tolerance toward a pro-inflammatory state that favors the onset or exacerbation of AITDs [7,9].

At the cellular level, the immunopathogenesis of AITDs involves an amplification feedback loop where Th1 and Th17 lymphocytes produce proinflammatory cytokines such as IFN-γ, TNF-α, and IL-6 [8,9,11]. Chronic stress-induced catecholamines activate the transcription factor nuclear factor kappa B (NF-κB), while GR resistance prevents cortisol from effectively suppressing these inflammatory pathways [7,9]. The resulting inflammatory milieu is characterized by an imbalance between Th17 and regulatory T cells (Treg), alongside increased oxidative stress and regulated cell death pathways such as apoptosis and pyroptosis [9,11].

Given the central role of immune and endocrine regulation in thyroid autoimmunity, clarifying the relationship between stress and AITD is clinically and conceptually relevant [12,13]. Numerous observational and interventional studies have examined associations between psychological stress and HT, GD, thyroid autoantibodies, disease relapse, and related psychological outcomes [[14], [15], [16], [17]]. However, the evidence remains fragmented, methodologically heterogeneous, and dispersed across different clinical phenotypes and stress-related constructs [18,19]. Previous reviews have explored this relationship [20], but the rapid expansion of the literature and the persistent methodological inconsistencies necessitate an updated, structured, and rigorous systematic integration of these findings.

A recent narrative review by Petrowski and Kahaly [21] offers a comprehensive account of how diverse physical, environmental, and psychological stressors affect hypothalamic–pituitary–thyroid axis function, drawing on animal and human evidence. However, whereas Petrowski and Kahaly synthesize the full spectrum of physical and psychological stressors across species, the present systematic review concentrates specifically on psychosocial stress in humans, applying a phenotype-specific lens that distinguishes Graves' disease from Hashimoto's thyroiditis and differentiates objective stressor exposure from subjective stress-response measures. Notably, the evidentiary bases of the two reviews show minimal overlap, indicating that the present review consolidates a substantially distinct body of primary literature on psychosocial stress and thyroid autoimmunity.

Hence, the primary objective of the present systematic review was to characterize phenotype-specific associations between psychosocial stress and AITD in Hashimoto's thyroiditis and Graves' disease, distinguishing objective stressor exposure from subjective stress response and identifying vulnerability factors that may shape this relationship. Secondary objectives included summarizing proposed mechanisms, assessing whether stress intensity is associated with differential effects, and evaluating the methodological quality and risk of bias of the included studies.

## 2 Methods

This systematic review was conducted in accordance with PRISMA guidelines, following a structured process including literature search, study selection, data extraction, quality assessment, and qualitative synthesis. Institutional ethics board approval was not required. The review protocol was registered in PROSPERO (https://www.crd.york.ac.uk/PROSPERO/view/CRD420251245470).

### 2.1 Information sources and search strategy

A comprehensive search was conducted in the databases PubMed, Embase, Scopus, and PsycINFO. The strategy employed an iterative process of refinement for each database. While the core concepts focused on 'stress' and ‘autoimmune thyroid disease’, search strings were expanded based on each database's strengths; for instance, including constructs such as perceived stress, chronic stress, and stressful life events in PsycINFO and Embase. In databases with broad biomedical coverage (Embase and Scopus), terms were restricted to title, abstract, and keywords to ensure the retrieval focused on psychological or psychosocial stress rather than biological or cellular mechanisms. Controlled vocabulary (e.g., MeSH terms in PubMed) was used where applicable. The full search strings for each database are provided in the Supplementary Material (Table S1).

Searches were limited to peer-reviewed articles published in English. No restrictions were applied regarding publication date. In addition, the reference lists of included studies were screened to identify further relevant publications.

### 2.2 Study selection

Study selection was conducted in two stages. First, two reviewers independently screened the titles and abstracts retrieved from the searches. Articles considered potentially eligible underwent full-text assessment, also performed independently by two reviewers. Screening decisions were based on predefined eligibility criteria. Disagreements were resolved through discussion, with involvement of a third reviewer when necessary. The selection process was documented using a PRISMA flow diagram.

### 2.3 Eligibility criteria

This review included studies involving human participants of any age, encompassing children, adolescents, and adults, regardless of sex, socioeconomic status, or geographic location. Eligible studies were required to examine the relationship between psychosocial stress and AITD, considering either stress or disease status as exposure or outcome, depending on the study design.

Stress could be assessed through validated psychometric instruments, clearly defined stressful conditions or life events. Comparator groups included individuals with lower or no exposure to stress, or individuals without AITD, as appropriate to the study design.

Studies were required to report at least one outcome of interest, including: incidence or presence of AITD (HT or GD); alterations in thyroid hormone levels; thyroid autoantibodies; or molecular and genetic markers related to thyroid autoimmunity.

Studies were excluded if they involved animals, in vitro or cell culture models, or if they did not assess psychological or psychosocial stress or did not include any thyroid-related outcomes. Studies lacking a comparator group, as appropriate to the design, were also excluded. Additionally, review articles, systematic reviews, meta-analyses, letters, commentaries, conference abstracts, case reports, and case series were excluded. Manuscripts without an available abstract, not published in peer-reviewed journals, or written in languages other than English were also excluded.

### 2.4 PI(E)CO strategy

Because the literature includes studies in which psychological stress is examined as a predictor of AITD, as well as studies in which AITD is considered the exposure influencing stress-related outcomes, a flexible PI(E)CO framework was adopted.

The population (P) included human participants of any age. The exposure (I/E) could be either psychological stress, assessed through validated psychometric instruments or clearly described stressful conditions or life events, or the presence of AITD. Comparator groups (C) included individuals with lower or no exposure to stress, or without AITD, as appropriate for each study design.

The outcomes (O) included the incidence or presence of AITD, specifically HT and GD. Additional outcomes included alterations in thyroid hormone levels, thyroid autoantibodies, and molecular or genetic markers related to thyroid autoimmunity or tissue injury. In studies where AITD was considered the exposure, outcomes could also include psychological or stress-related variables, such as perceived stress, anxiety, depressive symptoms, or other indicators of emotional distress.

### 2.5 Types of studies

The review included controlled study designs that allowed comparison between exposed and unexposed groups, including randomized clinical trials, cohort studies, case–control studies, cross-sectional studies with comparators, and controlled quasi-experimental studies.

### 2.6 Data extraction

From each included study, the following information was extracted: first author, year of publication, country, study design, population characteristics, comparator groups, exposure assessment, outcomes, and main results. Additional data items included sample size, sex distribution, and age range of participants.

Given the diversity of study designs, both psychological stress and AITD could be treated as either exposure or outcome variables. Accordingly, for each study, we recorded which variable was considered the exposure and which was considered the outcome.

Regarding exposure, the type of psychosocial stress assessed, the measurement method (e.g., validated psychometric scales, stress biomarkers, or clearly described stressful events), and the timing of assessment relative to outcomes were recorded. When AITD was treated as the exposure, disease characteristics were also documented. For outcomes, data related to the diagnosis of AITD (HT and GD) were extracted, as well as secondary outcomes such as thyroid hormone levels, autoantibody titers, and molecular or genetic markers associated with thyroid autoimmunity. When stress-related variables were considered outcomes, measures such as perceived stress or other indicators of emotional distress were also recorded. Whenever available, effect estimates reported by the authors were extracted, including measures of association (e.g., odds ratios, relative risks, correlation coefficients).

### 2.7 Quality assessment of studies

The methodological quality of included studies was assessed using the Effective Public Health Practice Project (EPHPP) Quality Assessment Tool for Quantitative Studies [22], which allows the appraisal of a broad range of study designs, including randomized controlled trials, cohort, case–control, and cross-sectional studies (Armijo-Olivo et al., 2012). The EPHPP tool evaluates studies across key domains, including selection bias, study design, confounders, blinding, data collection methods, and withdrawals and drop-outs. Each domain is rated as strong, moderate, or weak according to standardized criteria, and an overall global rating is assigned to each study. Quality assessment was conducted independently by two reviewers. Disagreements were resolved through discussion, with involvement of a third reviewer. The results of the quality assessment were not applied as exclusion criteria.

### 2.8 Data synthesis

A qualitative synthesis was conducted for all included studies, summarizing study characteristics, types of stress exposure, thyroid-related outcomes, and the direction and magnitude of reported associations. Patterns across studies were examined, including the consistency of findings, methodological strengths and limitations, and potential sources of heterogeneity.

A meta-analysis using a random-effects model was initially planned if sufficient homogeneity across studies was observed. A subset of five studies examining GD with comparable case–control designs [15,17,[23], [24], [25]] was considered for quantitative synthesis. However, substantial heterogeneity was identified (I^2^ > 99%), precluding meaningful quantitative pooling. Consequently, the findings were synthesized narratively.

### 2.9 Generative artificial intelligence disclosure

Chat PDF AI and Google NotebookLM were employed for data extraction from the included articles and Open AI Chat GPT-5.0 was used during manuscript preparation to enhance clarity and readability. Data extraction was supervised. The authors carefully reviewed and edited the AI generated texts as needed and take full responsibility for the content of the manuscript.

## 3 Results

The identification stage of the search yielded a total of 150 records across the selected databases (PubMed, Embase, Scopus, and PsycINFO). After removal of 42 duplicates, 108 records remained for screening. Following title and abstract screening, 60 records were excluded. Of the 48 reports sought for retrieval, 46 were assessed for eligibility, with two reports not retrieved.

After full-text assessment, 21 studies were excluded due to ineligible study design, absence of outcomes of interest, or other reasons, resulting in a final sample of 25 studies included in this review. These investigations encompass a combined total of N = 117,034 participants. The study selection process is illustrated in the PRISMA flow diagram (Fig. 1).

**Fig. 1:**
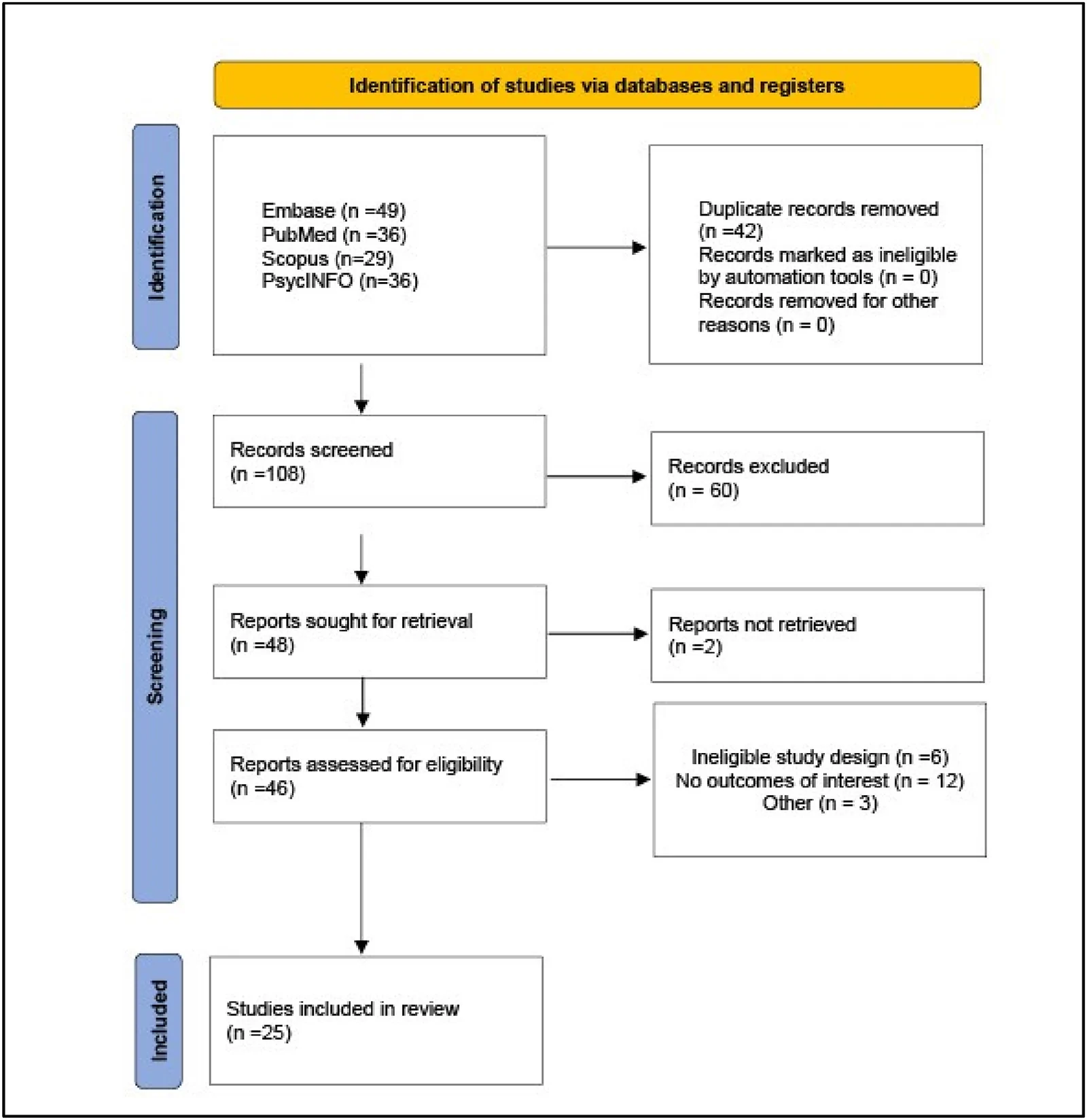
PRISMA flow diagram of study screening and selection.

All studies involved human participants and employed quantitative methodologies, including cross-sectional, case–control, cohort, and experimental designs. Across studies, stress exposure was assessed using validated psychometric instruments and structured interviews. Thyroid-related outcomes included the presence or incidence of AITD, thyroid function parameters, autoantibody levels, and, in some studies, molecular and genetic markers.

The included studies varied in their conceptual approach to the relationship between stress and AITD. Many examined psychological stress as an exposure variable, particularly in relation to the onset or progression of AITD (e.g., Ref. [14,[26], [27], [28], [29]]). A similarly substantial proportion of studies considered stress-related variables as outcomes (e.g., Ref. [18,19,[30], [31], [32], [33]]). This distribution reflects a relatively balanced representation of both perspectives within the literature.

Across the 25 included studies, stress was operationalized through diverse methodologies, reflecting the lack of a unified measurement standard in the field. The majority of investigations (15 studies) assessed exposure to stressful life events, using validated life-event inventories and structured interviews (e.g., Paykel's Interview for Recent Life Events, the Life Experiences Survey, the Hassles Scale) to quantify the occurrence of discrete negative life events [14,15,[17], [18], [19],[23], [24], [25], [26],^28^,^30^,^31^,^34^,^35^]. Subjective stress response and psychological burden were specifically evaluated in four studies using validated instruments such as the Perceived Stress Scale (PSS-10/14) [36] and the Depression Anxiety Stress Scale (DASS-21) [16,33,[37], [38], [39]]. Furthermore, three studies investigated early-life adversity through the Childhood Trauma Questionnaire (CTQ) [40] or Adverse Childhood Experiences (ACE) [41] screening tools [29,32,42]. The remaining studies employed either an experimental paradigm to measure acute neuroendocrine reactivity (one study) or non-validated dichotomous proxies for home and work stress exposure (two studies) [[43], [44], [45]].

The methodological quality of the included studies was initially assessed independently by two reviewers using the EPHPP tool. Discrepancies between their evaluations were identified and discussed, and consensus was achieved through consultation with a third reviewer with greater experience in the application of the instrument. Tables S2–S8 (Supplementary Material) present the reviewers’ final quality rating of the 25 studies. The final quality ratings classified 13 studies as strong, 8 as moderate, and 2 as weak, indicating that most included studies were of moderate to high methodological quality. Quality assessment was not possible for two studies due to the absence of full-text data.

The included studies were grouped into seven analytical categories, derived inductively from the reviewed literature to facilitate a structured synthesis of findings: (1) stress and hormones of the hypothalamic-pituitary-thyroid axis; (2) stress and overall autoimmune thyroid disease; (3) stress and Graves' disease; (4) stress and Graves' orbitopathy; (5) stress and Hashimoto's thyroiditis; (6) early-life stress and autoimmune thyroid disease; and (7) stress, autoimmune thyroid disease, and genetic factors.

The Results section presents seven tables summarizing the characteristics and main findings of the studies included in each analytical category. In addition to the tabulated data, a narrative synthesis is provided for each category, highlighting study designs, sample characteristics, and the main patterns observed in the association between stress and thyroid-related outcomes.

Notably, some studies focusing on the psychological burden of Hashimoto's thyroiditis, such as Müssig et al. [46] and Bianchi et al. [47], were not included in the review because they assessed psychological symptoms (e.g., anxiety, depression, or general distress) rather than stressor exposure or perceived stress as defined by the protocol's eligibility criteria. Similarly, while recent large-scale genomic analyses and Mendelian randomization studies provide critical evidence regarding the genetic substrate and causal architecture linking stress-related disorders to thyroid autoimmunity [48,49], they were omitted from the clinical narrative synthesis to maintain focus on direct observational stress measures. However, the biological insights regarding subjective burden and the genetic evidence of shared vulnerability from these studies are incorporated into the Discussion to substantiate the proposed conceptual framework.

### 3.1 Stress and hormones of the hypothalamic–pituitary–thyroid (HPT) axis

The three studies included in this category comprise a total of N = 250 participants, including n = 30 healthy women in an experimental setting [43], n = 114 women of reproductive age in a cross-sectional analysis [39], and n = 106 patients followed longitudinally after thyroidectomy [34] (Table S9). Evidence regarding the association between psychological stress and the HPT axis suggests that the thyroid's response is highly dependent on the nature of the stressor (acute vs. chronic) and the specific parameters of measurement.

In conditions of acute psychosocial stress, Fischer and colleagues [43] investigated immediate HPT activation in n = 30 healthy women using the Trier Social Stress Test (TSST) [50], a validated paradigm involving a mock job interview and mental arithmetic under pressure. The study identified a significant 36% surge in plasma TSH concentrations, which peaked 20 min after the onset of the stressor and returned to baseline by 110 min. Conversely, no acute changes were observed in T3 or T4 levels, suggesting that while acute psychosocial stress rapidly activates the pituitary, more sustained or severe stimuli may be necessary to impact peripheral thyroid hormone secretion [43].

Regarding perceived stress, cross-sectional findings failed to identify a significant relationship with thyroid parameters in healthy populations [39]. In an evaluation of n = 114 women of reproductive age using the Perceived Stress Scale (PSS-10), results showed no significant correlation between PSS scores and levels of TSH (r = 0.060, p = 0.529), T3, or T4 [39]. Furthermore, no significant differences were found in perceived stress levels when comparing women with normal thyroid function to those with subclinical hypothyroidism (SCH). The presence of SCH was instead found to be significantly associated with non-psychological factors, including older age and higher BMI [39].

The complexity of stress measurement is further highlighted by a prospective cohort study of patients following thyroidectomy [34]. The researchers compared multiple instruments, including the Daily Stress Inventory [51], the Social Readjustment Rating Scale [52], and the Stress Overload Scale-Short (SOSS) [53]. While TSH levels did not correlate with standardized daily or annual stress scales, they were significantly elevated in individuals classified as having “unconscious high-risk” stress. Identification of these stressors required an open-ended questionnaire to capture recent life events—such as bereavement or workplace changes—which standardized scales initially missed. These findings indicate that while global perceived stress measures often show no association, TSH may serve as a sensitive biomarker for recent stress overload, particularly when thyroid reserves are limited [34].

Taken together, the relationship between psychological stress and the HPT axis is characterized by acute neuroendocrine reactivity rather than consistent chronic baseline shifts as measured by global perceived stress scales [39,43]. While experimental psychosocial challenges can elicit an immediate and significant surge in TSH, static cross-sectional assessments often fail to identify significant correlations with thyroid parameters in healthy populations [39,43]. These findings suggest that the HPT axis exhibits a selective responsiveness to stress that is highly dependent on temporal dynamics and the individual's physiological context [34], providing a basis for understanding how stress-related changes influence specific autoimmune thyroid conditions.

### 3.2 Stress and overall autoimmune thyroid disease

This category synthesizes evidence from three studies involving a total of N = 1658 participants. This includes n = 1549 euthyroid individuals from two large-scale prospective and cross-sectional cohorts [18,19], and n = 109 participants in a study examining immune and oxidative stress markers [31] (Table 1). The findings are heterogeneous and suggest that the relationship between stress and thyroid autoimmunity is contingent upon the disease stage and the specific stress construct measured. While the two larger studies focused on euthyroid populations found no evidence that stress exposure triggers the initial development of thyroid autoantibodies [18,19], evidence from clinical populations indicates that the subjective negative burden of stress—rather than the mere frequency of events—correlates significantly with markers of disease activity and antibody titers [31].

**Table 1:** Details of studies examining the association between stress and autoimmune thyroid disease (AITD).

Authors/Location	Characteristics of the samples (n)	Sex (M/F) Age range (years)	Study design	Exposure/Intervention	Outcomes	Main findings
Strieder et al. [19] (Netherlands)	Euthyroid subjects: 759 Divided in two groups: • Thyroid peroxidase antibodies (TPO-Ab) positive: 183 • TPO-Ab negative: 576	All female TPO-Ab positive group: 39.7 ± 12 y TPO-Ab negative group: 34.2 ± 12 y	Cross-sectional	Exposure: Presence of TPO-Ab	Self-reported stress and mood measures: recent stressful life events (number and valence), daily hassles (frequency and intensity), and affect (positive and negative; PANAS^a^). Additional variables included demographic, clinical, and behavioral factors (age, TSH, free T4, education, employment status, and psychotropic medication use).	TPO-Ab–positive women were older and had higher TSH levels than TPO-Ab–negative women, with no differences in free T4 or other sociodemographic variables. No significant associations were found between TPO-Ab status and stressful life events, daily hassles, or mood measures after adjustment for age. Overall, the study found no evidence linking stress or affective factors to the presence or levels of thyroid autoantibodies in euthyroid women.
Effraimidis et al. [18] (Netherlands)	Euthyroid individuals with a positive family history of ATD: 790	All female 18-65 y	Prospective cohort (5-year follow-up)	Exposure: Onset of positive TPO-Ab, onset of overt hyper and hypothyroidism, during follow-up.	Prospective assessment of stress exposure using questionnaires on recent stressful life events, daily hassles, and mood (PANAS).	No difference in scores of perceived stress between patients who developed positive TPO-Ab, overt hyper or hypothyroidism as compared with those who did not (matched controls [2:1]). Hypothyroid cases reported fewer negative affective symptoms at diagnosis. Authors suggest that stress is not involved in the pathogenesis of AITD.
Vaivode et al. [31] (Latvia)	Hashimoto Thyroiditis (HT): 47 Graves Disease (GD): 13 Healthy controls: 49	HT group: (3/44) Median age: 41 y (27–50) GD group: (2/11) Median age: 41 y (29–57) Control group: (11/38) Median age: 30 y (26–46)	Cross-sectional	Exposure: Presence of ATD (HT or GD)	Stress profile based on the number and impact of life events over the last 12 months (positive/negative). Assessment of thyroid function parameters, autoantibody levels, plasma levels of cytokines, selenium, selenoprotein P and glutathione peroxidase (GPx) activity.	No differences were observed in the number of stressful events among HT, GD, and control groups, although HT patients showed a higher burden of negative stress. Stress measures were correlated with selenium-related markers (selenoprotein P and GPx activity) and TPO-Ab levels in disease groups. No differences were found in selenium concentrations or cytokine profiles between groups. Authors suggest that selenoproteins may serve as biomarkers in the relationship between stress and AITD.

a PANAS: Positive and Negative Affect Schedule.

Evidence from large-scale cross-sectional research suggests that neither stressor exposure nor the affective stress response is associated with the presence of thyroid autoantibodies in healthy populations. Strieder et al. [19] evaluated n = 759 euthyroid women—all relatives of AITD patients—to determine if stress was an early marker for Hashimoto's thyroiditis. The researchers assessed stressor exposure using the Recently Experienced Stressful Life Events (RESLE) questionnaire (60 items over the last 12 months) [54] and the Everyday Problem Checklist (114 items measuring daily hassles over the last 2 months) [55]. Additionally, they measured the stress response via the Positive and Negative Affect Schedule (PANAS) [56]. After adjusting for age—a significant confounder—no associations were found between TPO-Ab status and the number of life events, the intensity of daily hassles, or affective states [19].

Longitudinal findings further support this lack of association regarding disease pathogenesis. Effraimidis et al. [18] conducted two nested case–control studies within a 5-year prospective cohort of n = 790 euthyroid women. Using the same battery of instruments as Strieder (RESLE, Everyday Problem Checklist, and PANAS), the authors found that stress exposure was not significantly different between subjects who developed de novo TPO-Ab and matched controls. Furthermore, no differences in stress scores were observed at baseline, one year prior to, or at the time of diagnosis for those who progressed to overt hyper- or hypothyroidism [18]. These results led the authors to conclude that psychological stress is likely not involved in the initial pathogenesis of AITD in susceptible individuals.

Conversely, more recent cross-sectional data suggest that the subjective stress response may be more relevant than mere stressor exposure in patients already exhibiting autoimmunity. Vaivode et al. [31] compared patients with HT and GD to healthy controls using the Life Experiences Survey [57]. While there were no significant group differences in the total number of major life events (exposure), patients with HT reported a significantly higher negative stress level (subjective response/impact) compared to their positive stress levels [31]. Notably, significant positive correlations were found between TPO-Ab and Tg-Ab levels and multiple stress parameters, including the total number of events, events occurring 7–12 months prior, and the negative stress level [31].

Taken together, these findings suggest that while stress may not trigger the disease pathogenesis [18,19], the negative psychological burden is associated with biological markers of disease activity and oxidative stress in affected individuals [31]. The observation that patients report a higher negative burden of stress—which correlates with antibody titers [31]—highlights the importance of distinguishing between stressor exposure and the subjective stress response.

### 3.3 Stress and graves’ disease

The nine studies analyzed in this section encompass a total of N = 2033 participants, consisting of n = 1113 patients with GD and n = 920 healthy controls or clinical comparators (e.g., patients with toxic nodular goitre) across case–control and cohort investigations [14,15,17,[23], [24], [25], [26], [27], [28]]. Six studies had a case–control design and three were cohort studies. Across these investigations, the evidence consistently suggests a significant association between stressful life events—particularly those of a negative nature—and both the onset and clinical trajectory of the disease.

Case–control studies provide evidence that both stressor exposure (objective events) and the subjective stress response (perceived impact) are significantly higher in patients preceding the onset of GD. Winsa et al. [17] demonstrated in a population-based study that patients reported significantly more negative life events in the 12 months prior to diagnosis, with an odds ratio of 6.3 for those in the highest negative score category. Utilizing the Paykel's Interview for Recent Life Events [58], Radosavljevic et al. [23] found that GD patients reported a significantly higher mean number of stressful events compared to controls (M = 3.18 ± 0.23 vs. 1.85 ± 0.20, p = 0.0001). The authors identified specific triggers related to work (change in conditions, RR = 7.0; unemployment, RR = 8.0) and interpersonal conflicts (arguments with a superior, RR = 3.5; or with a partner, RR = 11.0). Similarly, Kung [15] and Matos-Santos et al. [25,27] utilized the Life Experiences Survey and the Hassles Scale [55] to show that GD patients not only experienced more negative events but also perceived them with significantly higher severity ratings compared to controls. These researchers highlighted a specific temporal window for these events, noting that most precipitating stressors occurred between 7 and 12 months prior to symptom onset. Furthermore, Yoshiuchi et al. [24] identified a marked sex-specific effect, where stressful life events were associated with a 7.7-fold increased risk of GD in women, whereas no significant association was identified in men.

Beyond disease onset, evidence from cohort studies indicates that stress significantly influences the prognosis and immunological activity of GD. Stewart et al. [26] found that patients exposed to major stressors in the 12 months preceding diagnosis became hypothyroid significantly earlier following radioactive iodine therapy (50% at 12 months vs. 36 months in non-stressed patients), suggesting that stress may potentiate the autoimmune response. Regarding pharmacological treatment, Fukao et al. [14] utilized Natsume's Stress Inventory [59] and Hayashi's Daily Life Stress Inventory [60] to show that major life event scores correlated significantly with TSH receptor antibody (TRAb) activity (r = 0.42, p < 0.001) and thyroid volume (r = 0.48, p < 0.001). The authors reported that patients who relapsed after antithyroid drug withdrawal experienced significantly higher daily hassles and exhibited personality traits, such as depression and psychasthenia, linked to a negative appraisal of stress.

The role of stress as a recurrent trigger was further emphasized by Vita et al. [28] in a 21-year follow-up study. The researchers observed that every episode of hyperthyroidism—whether onset, exacerbation, or relapse—was systematically preceded by at least one stressful event in susceptible individuals. Notably, the total number of stressful events correlated directly with the number of relapses (r = 0.49, p < 0.0001). Collectively, these findings support a conditional model where negative life events and a high subjective burden of stress act as precipitating factors that precipitate or amplify Graves’ hyperthyroidism in genetically predisposed individuals (see Table 2).

**Table 2:** Details of studies examining the association between stress and Graves’ disease (GD).

Authors/Location	Characteristics of the samples (n)	Sex (M/F) Age range (years)	Study design	Exposure/Intervention	Outcomes	Main findings
Stewart et al. [26] (Canada)	GD patients referred to a nuclear medicine unit for radioactive iodine therapy: 293 (with clinical outcomes compared between stressed and non-stressed subgroups)	(59/234) Median age at diagnosis: 37 y (range 13-78 y)	Retrospective cohort	Exposure: Presence of stressful life events in the 12 months preceding GD symptom onset.	Thyroid status during follow-up (euthyroid or hypothyroid; 1–155 months), time from initial treatment with radioactive iodine to hypothyroidism, and number of treatments required (single vs. multiple).	A total of 27.6% of patients reported a stressful event within 12 months prior to symptom onset. Those exposed to stress developed hypothyroidism significantly earlier after radioactive iodine treatment (50% at 12 months vs. 36 months in non-stressed patients; p < 0.01) and had lower long-term rates of euthyroidism (5% vs. 17% at 10 years). Stress was associated with reduced probability of remaining euthyroid (p = 0.01). Authors propose that recent stress may potentiate radioiodine-induced hypothyroidism, possibly via enhanced autoimmune activity.
Winsa et al. [17] (Sweden)	Newly diagnosed GD: 208 Healthy controls: 372	Not reported	Case-control	Exposure: Presence or absence of GD	Assessment of stressful life events, family history of thyroid disease, social support, marital status, occupation, lifestyle factors (smoking, alcohol use, physical activity), and personality traits, collected via a mailed questionnaire.	Patients with GD reported significantly more negative life events in the 12 months preceding diagnosis compared to controls (OR = 6.3 for the highest negative score category). A positive family history of thyroid disease was also associated with increased risk (OR = 3.6). Additionally, patients were more likely to be divorced (OR = 1.8) and reported lower alcohol consumption (OR = 0.4).
Kung [15] (Hong Kong)	Newly diagnosed GD: 95 Healthy controls: 95	(15/80, in each group) 33 ± 1 y	Case-control	Exposure: Presence or absence of GD	Number of stressful life events in the previous 12 months.	Patients with GD reported significantly more negative life events (P < 0.0005) and greater perceived impact, resulting in higher negative event scores (P < 0.0001) compared to controls. They also experienced more daily hassles and higher hassle scores (both P < 0.001). In contrast, the coping strategies, were similar between groups.
Radosavljevic et al. [23] (Serbia)	Newly diagnosed GD: 100 Healthy controls: 100	(7/93, in each group) GD group: 40.6 ± 9.8 y Control group: 40.6 ± 2 y	Case-control	Exposure: Presence or absence of GD	Assessment of stressful life events occurring in the 12 months preceding the diagnosis of GD in patients, or 12 months before the interview date for controls. Paykel's Interview for Recent Life Events (covering 61 life events) was administered to all participants.	Patients with GD reported a significantly higher mean number of stressful life events in the 12 months preceding diagnosis compared to controls (3.18 ± 0.23 vs. 1.85 ± 0.20; p = 0.0001). Several specific stressors were more prevalent among patients, particularly work-related changes (RR up to 7.0), unemployment (RR = 8.0), interpersonal conflicts (including with spouse, RR = 11.0), hospitalization of a family member (RR = 3.25), and financial difficulties (RR = 3.25).
Yoshiuchi et al.[24] (Japan)	Newly diagnosed GD: 228 Healthy controls: 228	(46/182, in each group) Adults	Case-control	Exposure: Presence or absence of GD.	Number of stressful life events (SLE). History of smoking, drinking habits, social support and coping skills also assessed.	After adjustment for potential confounders (smoking, alcohol use, social support, and coping), SLE were significantly associated with increased risk of GD in women (RR = 7.7; 95% CI: 2.2–27; p < 0.001). Smoking was also independently associated with risk (RR = 5.1; 95% CI: 1.0–27; p < 0.001). No significant associations were observed in men, indicating a sex-specific effect.
Matos-Santos et al. [25] (Portugal)	GD: 31 Toxic Nodular Goitre (TNG): 31 Healthy controls group (CG): 31	(9/22, in each group) GD: 38.4 ± 10.9 y TNG: 48.3 ± 11.1 y CG: 41.1 ± 11.8 y	Case-control	Exposure: Presence or absence of GD. Presence or absence of TNG.	Stressful life events (SLEs) in the 12 months preceding symptom onset were assessed using the Life Experiences Survey (LES), including number and perceived impact (negative, positive, and neutral). Outcomes included comparisons of SLE measures across GD, TNG, and control groups, as well as calculation of odds ratios.	Patients with GD reported a significantly higher number and greater impact of stressful life events (SLEs), particularly negative events, compared to both toxic nodular goitre (TNG) and control groups (P < 0.001). No differences in SLE measures were observed between TNG and controls. Most stressful events occurred 7–12 months prior to disease onset. Authors suggest that SLEs may act as precipitating factors for GD, but not for TNG.
Matos-Santos et al. [27] (Portugal)	GD (diagnosed within the last 12 months: 31 Healthy controls:31	(9/22, in each group) GD group: 38.5 ± 10.9 y Healthy controls: 41.1 ± 11.8 y	Case-control	Exposure: Stressful life events (SLE) experienced in the 12 months preceding the onset of thyroid disease symptoms for the GD group. The control group was evaluated for SLEs over a comparable period.	The number and impact (positive, negative, neutral) of SLEs reported by participants. The Life Experiences Survey (LES) was used.	Patients with GD reported a significantly higher number and greater impact of negative SLEs compared to controls (p < 0.001), while no differences were observed for positive or neutral events. Most events occurred 7–12 months prior to disease onset and were commonly related to work, health, and changes in daily activities. Authors argue that negative SLEs may act as precipitating factors for GD, possibly through stress-related immunological mechanisms.
Fukao et al. [14] (Japan)	GD patients treated with antithyroid drugs who were euthyroid after 2-5 years of therapy: 69 Healthy controls: 32	GD Patients (4/65) and Healthy controls (1/31) Relapsed GD subgroup (n = 41): 39.3 ± 14.6 y Remitted GD subgroup (n = 28): 43.4 ± 12.4 y Healthy controls: 36.7 ± 12.6 y	Prospective cohort (Once included in the study, patients underwent withdrawal of antithyroid medication and were followed for 12 months; those remaining euthyroid were classified as ‘remitted GD’, while those relapsing within 12 months were classified as ‘relapsed GD’.	Exposure: Stressful life events and personality traits (using the MMPI^a^ measure)	Relapse or remission of GD after cessation of antithyroid drug therapy; correlations between stress, psychological traits, and clinical markers (TSH receptor antibodies, thyroid volume).	Higher stress from major life events was significantly correlated with TSH receptor antibody activity and thyroid volume. Relapsed patients showed greater levels of daily hassles and a higher prevalence of personality traits such as hypochondriasis, depression, paranoia, and psychasthenia. Given these results, the authors suggest that stress coping strategies may help improve outcomes in GD.
Vita et al. [28] (Italy)	Patients with GD treated pharmacologically:58/ a Note: personal history of smoking, thyroidectomy and radioiodine therapy, as well as ATD in family members were exclusion criteria	(22/36) 35.4 ± 14.6 y	Retrospective cohort	Exposure: Number of stressful life events.	Relapse of GD. Each participant was followed-up for at least 5 years after the withdrawal of pharmacologic treatment.	Patients who relapsed had significantly more stressful life events in the previous 12 months as compared with those who did not. The number of stressful life events was positively associated with the number of relapses. The younger the patients, the shorter the interval between the stressful event and Grave's disease relapse.

a MMPI: Minnesota Multiphasic Personality Inventory.

### 3.4 Stress and graves’ orbitopathy (GO)

Two investigations involving a total of N = 2806 participants examined the role of stress in GO. The sample includes n = 1602 patients with GO and 1000 patients with GD without orbital involvement [45], alongside n = 204 control subjects with other autoimmune conditions [30]. These studies evaluated the impact of psychological stress on the condition, identifying it as both a potential risk factor for orbital manifestations and a significant contributor to patients' psychological burden [30,45] (Table 3).

**Table 3:** Details of studies examining the association between stress and Graves’ orbitopathy (GO).

Authors/Location	Characteristics of the samples (n)	Sex (M/F) Age range (years)	Study design	Exposure/Intervention	Outcomes	Main findings
Kahaly et al. [30] (Germany)	GO: 102 Type 1 diabetes (T1D): 102 Inflammatory bowel diseases (IBD): 102	(12/80, in each group) 18–72 y (median 41 y)	Retrospective cohort	Exposure: Being a patient diagnosed with one of the autoimmune conditions.	Assessment of quality of life (QoL) (SF-36^a^), coping styles, emotional distress (symptoms of anxiety and depression), and stressful life events.	A high prevalence of stressful life events was observed among GO patients, with 72% reporting at least one event in the previous 6 months. Stressful events were associated with impaired (QoL) and showed significant (p = 0.001) positive correlations with anxiety (r = 0.33) and depression (r = 0.32). Greater stress also linked to disease severity, with higher numbers of events and more negative life change scores observed in patients with optic neuropathy and active GO.
Zhou et al. [45] (China)	Graves' disease (GD) with GO: 1500 GD patients without orbitopathy: 1000	(70%/30%, in each group) Participants with GO - mean age at the onset of GD: 29 ± 5.2 y Participants without GO - mean age at the onset of GD: 28 ± 4.3 y	Retrospective	Exposure: Being a patient diagnosed with GD and GO.	Patients' charts were reviewed to extract data on smoking status, stress (dichotomic assessment), family history of thyroid disease, levels of free T4 and free T3, anti-thyroid antibodies, as well as age and body mass index.	GO was associated with the presence of stress, with a higher prevalence of smoking, and a greater frequency of family history of thyroid disease. Patients with GO also exhibited higher levels of free T4, free T3, TSH receptor antibodies, and TPO-Ab, as well as a higher proportion of overweight individuals, larger thyroid gland size, and increased rates of drug-induced hypothyroidism.

a SF-36: 36-Item Short Form Health Survey.

Stressor exposure in the home and work environment was investigated in a large retrospective study of n = 2500 Chinese patients. Zhou et al. [45] found that both stress at home (31% vs. 11%, p < 0.0001) and a stressful work environment (32% vs. 4%, p < 0.001) were significantly more prevalent in patients with GO compared to those with GD without orbital involvement. These findings suggest that chronic environmental stressors are independent predictors for the clinical manifestation of the disease.

Complementary to this, Kahaly et al. [30] focused on the subjective stress response and psychosocial morbidity in a prospective study using the Life Experience Survey and the Hospital Anxiety and Depression Scale (HADS) [61]. While 86% of patients reported at least one stressful event in the six months preceding diagnosis, the negative life change score (subjective impact) was significantly higher in patients with active GO and optic neuropathy. These negative impact scores correlated with higher levels of anxiety (r = 0.33, p < 0.001) and depression (r = 0.32, p < 0.001) and were identified as major determinants of impaired quality of life.

Collectively, these findings indicate that stress in GO acts as both a precipitating factor and a psychological outcome, where the subjective negative burden—rather than objective exposure—shows a significant correlation with disease severity and visual impairment [30,45].

### 3.5 Stress and Hashimoto's thyroiditis

The three studies included in this subsection involved a total of N = 305 participants, comprising n = 228 patients with HT or newly diagnosed primary hypothyroidism and n = 77 healthy controls [16,33,38] (Table 4). Case–control and randomized controlled trial (RCT) designs were applied. Overall, the evidence indicates that patients with HT exhibit a high subjective stress response and altered autonomic regulation, while interventional data suggest that reducing psychological burden may modestly influence immunological markers, specifically through the reduction of antithyroid antibody titers.

**Table 4:** Details of studies examining the association between stress and Hashimoto's thyroiditis.

Authors/Location	Characteristics of the samples (n)	Sex (M/F) Age range (years)	Study design	Exposure/Intervention	Outcomes	Main findings
Markomamolaki et al. [16] (Greece)	Hashimoto Thyroiditis: 60 Divided in two groups: • Stress management group: 30 • Standard care: 30	All female Intervention group: 45.7 ± 12 y Control group: 46.8 ± 12 y anos	Intervention study (two-arm parallel group, non-blind, randomized controlled trial)	Intervention: An 8-week stress management program with weekly sessions, including diaphragmatic breathing, progressive relaxation, cognitive restructuring, dietary adjustments	Levels of TPO-Ab, Tg-Ab antibodies, and TSH; Body Mass Index (BMI); Depression, Anxiety, and Stress (DASS-21^a^) scores; Healthy Lifestyle (HLPCQ^a^) scores	The intervention led to significant reductions in Tg-Ab levels (p = 0.01), while decreases in TPO-Ab and TSH were not statistically significant. Significant improvements were observed in stress (p < 0.001), depression (p = 0.01), and anxiety (p = 0.01), along with increased healthy lifestyle scores (p < 0.001), which showed the strongest effect. No significant changes were found in BMI.
Singh et al. [33] (India)	Newly diagnosed hypothyroidism: 77 Healthy controls: 77	(11/66, in each group) 33 ± 5 y for both groups	Case-control	Exposure: Presence or absence of Hypothyroidism.	Perceived stress was assessed using the Perceived Stress Scale (PSS-14), along with heart rate variability (HRV). Thyroid hormone levels, anthropometric measures, and vital signs were also evaluated.	Hypothyroid patients exhibited higher perceived stress than controls, with a greater proportion classified as high stress. They also showed altered autonomic function, including lower heart rate, reduced HRV indices (RMSSD, pRR50), lower high-frequency power, and increased LF/HF ratio, indicating sympathovagal imbalance. TSH levels correlated strongly with LF/HF ratio (r = 0.781) and moderately with perceived stress.
Macarenco et al. [38] (Romania)	Hashimoto's thyroiditis (HT): 91 Three subgroups: • Eye movement desensitization and reprocessing (EMDR) group: 30 • Placebo group: 30 • Treatment-as-usual (TAU) group: 31	EMDR group: (2/28) 40 ± 9 y Placebo group: (0/30) 41 ± 8 y TAU group: (0/31) 41.7 ± 8 y	Randomized controlled trial	Intervention groups included EMDR plus TAU, placebo plus TAU, and TAU alone. EMDR, a psychotherapy for psychological trauma, targeted distressing memories predating illness onset. The placebo condition avoided trauma-focused content, and TAU consisted of standard care.	Biological markers (TPO-Ab and TG-Ab levels) and psychological measures, including depression, anxiety, and stress (DASS-21), dissociation (DES^a^), alexithymia (TAS-20^a^), trait anger (STAXI-2^a^), emotion regulation (DERS-18^a^), and quality of life (WHOQOL-BREF^a^); trauma history (MAES^a^) was assessed at baseline.	EMDR was associated with significant improvements in multiple psychological outcomes (e.g., dissociation, depression, anxiety, stress, trait anger, emotion regulation), which were maintained at 3-month follow-up and generally exceeded those of placebo and TAU groups. TPO-Ab levels decreased in the EMDR group, although between-group differences at follow-up were not significant after multiple comparison correction. No changes were observed in Tg-Ab levels.

a DASS-21: Depression, Anxiety and Stress Scale-21/HLPCQ: Healthy Lifestyle and Personal Control Questionnaire/DES: Dissociative Experiences Scale/TAS-20: Toronto Alexithymia Scale-20/STAXI-2: State-Trait Anger Expression Inventory-2/DERS-18: Difficulties in Emotion Regulation Scale-18/WHOQOL-BREF: World Health Organization Quality of Life Instrument-Abbreviated version/MAES: Maltreatment Abuse and Exposure Scale.

Evidence from clinical populations highlights a significant link between perceived stress and autonomic dysfunction in hypothyroid patients, a common clinical manifestation of HT. Singh et al. [33] evaluated n = 77 newly diagnosed patients using the Perceived Stress Scale (PSS-14) to measure the subjective stress response and Heart Rate Variability (HRV) as a physiological marker of the autonomic response. Patients reported significantly higher PSS scores compared to healthy controls (M = 25.82 ± 2.8 vs. 22.47 ± 2.1, p < 0.001), with the majority (54.5%) classified as experiencing high stress levels. Furthermore, these patients exhibited a marked sympathovagal imbalance, characterized by lower high-frequency power and a significantly higher LF/HF ratio, which correlated strongly with TSH levels (r = 0.78, p < 0.001). These findings suggest that higher perceived stress is associated with both the biochemical severity of hypothyroidism and altered cardiac autonomic control.

Interventional studies provide preliminary support for the role of stress management in modulating the autoimmune response. Markomanolaki et al. [16] conducted an 8-week RCT in women with HT, implementing a program that included diaphragmatic breathing, progressive relaxation, and cognitive restructuring. The intervention led to significant improvements in the affective stress response—measured via the DASS-21—and a significant reduction in Tg-Ab titers (p = 0.01). While TPO-Ab and TSH levels showed a downward trend, these changes did not reach statistical significance.

The impact of resolving past stressor exposure was further explored by Macarenco et al. [38] in a three-arm RCT using Eye Movement Desensitization and Reprocessing (EMDR) [62]. The protocol focused on reprocessing distressing memories predating the illness, assessed through the Maltreatment Abuse and Exposure Scale (MAES) [63]. EMDR led to sustained improvements in psychological traits associated with chronic stress, including significant reductions in alexithymia (TAS-20) [64] and dissociation (DES) [65], alongside improvements in depression and anxiety. Although within-group analysis showed a significant reduction in TPO-Ab levels in the EMDR group from baseline to follow-up, between-group differences were not sustained after multiple comparison corrections.

In summary, HT is consistently associated with an increased psychological burden and a heightened subjective stress response, which correlates with biochemical and autonomic markers of the disease [33]. While the direct role of stress as a causal factor for disease onset remains less clear than in GD, interventional findings suggest that addressing both current stress levels and past traumatic exposures can improve psychological well-being and may have a selective, albeit modest, influence on thyroid autoantibody activity [16,38].

### 3.6 Early-life stress and autoimmune thyroid disease

Three studies investigated early-life adversity in a total of N = 109,206 participants. This scale is primarily driven by two massive population-based cohorts involving n = 108,915 individuals (both investigated by Ref. [29]), supplemented by two clinical case–control studies involving n = 291 women evaluated for thyroid autoimmune phenotypes [32,42] (Table S10). Since these investigations focused on stressor exposure during sensitive developmental periods [2], the evidence suggests a dose–response relationship between early adversity and adult thyroid autoimmunity primarily in women.

At the population level, Köhler-Forsberg et al. [29] provided the most robust evidence using two large cohorts: the Icelandic SAGA (n = 22,423 women) and the UK Biobank (n = 86,492 women). Stressor exposure was retrospectively assessed using the Childhood Trauma Screener-5 items (CTS-5) [66] and a modified ACE International Questionnaire (ACE-IQ) [67]. The study identified a significant dose–response association, where every additional ACE was linked to a 10% to 11% increase in the prevalence of thyroid disease (PR = 1.11, 95% CI 1.10–1.13 in SAGA; PR = 1.09, 95% CI 1.08–1.11 in UKB). Notably, sexual abuse, physical neglect, and emotional neglect were the specific stressors most consistently associated with thyroid disease. Furthermore, causal mediation analysis revealed that approximately 25% of this association was mediated through adult mental distress (symptoms of depression, anxiety, and PTSD), highlighting how early exposure shapes the long-term subjective stress response.

Focusing specifically on the autoimmune phenotype in women, Corso et al. [32] conducted a case–control study (n = 208) using the Childhood Trauma Questionnaire. The findings indicated that female patients with AITD were significantly more likely to report emotional forms of maltreatment compared to matched controls. Specifically, patients reported a higher frequency of emotional neglect (29.7% vs. 19.5%, p = 0.051) and emotional abuse (41.3% vs. 32%, p = 0.091). The prevalence of emotional abuse in the AITD group was more than three times higher than in age-matched norm samples.

The biological link between early adversity and specific autoantibody activity was further clarified by Aydogan et al. [42]. In a study of n = 52 patients with HT, the researchers utilized the Turkish version of the CTQ [68] and found that the emotional neglect score was significantly higher in the HT group (M = 10.79 ± 3.90) than in healthy controls (M = 9.13 ± 3.22) (p = 0.04). A significant positive correlation was identified between the childhood emotional neglect score and TPO-Ab levels (r = 0.26, p = 0.02).

Taken together, these findings indicate that early-life stress—particularly emotional neglect and abuse—is a significant, albeit modest, risk factor for the development of AITD. These early exposures appear to create a biological vulnerability, possibly through chronic HPA-axis dysregulation or “wear and tear” on the immune system [2], which increases the likelihood of thyroid autoimmunity later in life [29,32,42].

### 3.7 Stress, autoimmune thyroid disease, and genetic factors

The relationship between stress and genetic factors was evaluated in two studies with a combined total of N = 776 participants. The sample includes n = 646 patients with GD (with and without orbitopathy) and n = 130 euthyroid controls utilized for HLA typing and gene–environment interaction analysis [35,44] (Table S11). The findings support a “vulnerability model,” where the impact of stressor exposure on thyroid function is contingent upon the individual's genetic architecture.

Evidence regarding the role of the Human Leukocyte Antigen (HLA) region indicates that specific alleles modulate the clinical expression of stress-triggered GD. Vita et al. [35] conducted an observational study of n = 58 Caucasian patients whose hyperthyroidism onset or recurrence was preceded by at least one stressor exposure (psychological, infectious, or physical) within a 12-month window. The researchers identified that five HLA alleles—B8, Cw7, DQ2, DR3, and DR4—were significantly more frequent in patients with stress-related GD compared to healthy controls (OR ranging from 2.1 to 5.6). Notably, the genetic profile influenced the biochemical severity of the disease; for instance, carriers of the Cw7 allele exhibited the highest fold increases in serum FT3 at onset. Furthermore, the study demonstrated that HLA typing could predict outcomes after antithyroid drug withdrawal, as the Cw7 allele was significantly over-represented in patients who experienced exacerbations or relapses (p = 0.05).

The interplay between environmental stress and genetic polymorphisms was further explored in the context of GO. Yang et al. [44] investigated a Chinese Han population (n = 588) to assess how single nucleotide polymorphisms (SNPs) within the LFA-1/ICAM-1/GSK-3β pathway interact with environmental hazards. Using a dichotomic assessment of stressor exposure, the study found that both a stressful work environment (OR = 1.64, p = 0.019) and stress at home were independent predictors of GO risk. Crucially, the researchers identified significant gene-environment interactions (GEI), where specific SNPs (such as rs12716927, rs5498, and rs6438552) significantly modified the influence of work and home stress on GO susceptibility. These results indicate that environmental stress does not act in isolation but synergizes with immunological pathways to trigger inflammatory manifestations in the orbit.

In summary, the evidence from genetic and gene–environment interaction studies suggests that stress-related factors and AITD share complex biological substrates. These findings reinforce the view that the effect of stress is conditional, appearing most pronounced when individuals carry specific genetic risk factors that facilitate a maladaptive immunological response to environmental demands.

## 4 Discussion

This systematic review, integrating data from over N = 117,000 individuals, demonstrates that the relationship between psychological stress and AITD is clinically relevant yet dependent on the clinical phenotype and the specific stress construct evaluated [2,69]. Our consolidated findings reveal a nuanced pattern where GD exhibits a robust association with stressful life events, preceding disease onset and recurrence [15,17,23,28], whereas HT is more closely linked to a heightened subjective stress response and indicators of psychological distress in already diagnosed patients [31,33]. Furthermore, evidence of acute neuroendocrine reactivity in the pituitary [43] and the identified dose–response relationship between childhood adversity and adult thyroid autoimmunity [29,42] suggest that stress operates as a complex biological modulator rather than a simple trigger. Ultimately, these data support a conditional model in which the influence of stress on thyroid autoimmunity is the result of a dynamic interaction between exposure timing, the magnitude of the subjective psychobiological burden, and the individual's genetic architecture [29,35,44].

### 4.1 Phenotype-dependent associations: graves' vs. Hashimoto's

The link between stress and thyroid autoimmunity is highly phenotype-dependent, aligning with the specific immunological and biochemical hallmarks of each condition. In GD, the evidence reveals a consistent pattern where exposure to stressful life events precedes disease onset and relapses [15,17,23]. While clinical GD is characterized by thyrotoxicosis and suppressed TSH levels, experimental evidence in healthy individuals indicates that the HPT axis is dynamically responsive to situational overload, manifesting as a rapid, transient TSH surge [43]. Although the direct role of this surge in GD pathogenesis remains to be longitudinally established, it demonstrates that psychological stress elicits immediate neuroendocrine shifts. More robustly, the triggering of GD in susceptible individuals is likely mediated through sympathetic activation and the release of pro-inflammatory cytokines (e.g., IL-6, TNF-α), which can activate the NF-κB pathway and facilitate the breakdown of immunological tolerance [7,69].

In HT, stress is closely associated with disease activity and autonomic dysfunction, as evidenced by the marked sympathovagal imbalance—characterized by a high LF/HF ratio—observed in these patients [33]. Within this phenotype, the sustained subjective stress response may be biologically reinforced by the chronic inflammatory milieu characteristic of the disease [8,9,11]. While HT leads to lymphocytic destruction, it also fosters the systemic circulation of pro-inflammatory cytokines such as IL-6 and TNF-α [8,9]. According to the psychoneuroimmunology framework, these cytokines can signal the central nervous system to induce a coordinated set of behavioral changes known as ‘sickness behavior’, including lethargy and heightened emotional reactivity [7,69]. This biological pathway explains why TPO-Ab positivity serves as a significant predictor of diminished psychosocial well-being over time, even after biochemical euthyroidism is achieved [46,47,70]. Thus, in the HT phenotype, stress is not merely an external precipitant, but a self-perpetuating psychological burden sustained by the underlying autoimmune activity, functioning as a feedback loop between systemic inflammation and the subjective stress response [7,69].

### 4.2 Interpretation of heterogeneity through measurement specificity

A primary limitation in research concerning stress and AITD is the historical lack of specificity in defining and measuring the stress construct [2]. As indicated by our results, the high degree of heterogeneity observed across the literature is best explained by the critical methodological distinction between stressor exposure—the objective occurrence of environmental events—and the subjective stress response, which encompasses the individual's perceived psychological and biological burden [2]. When this distinction is neglected, the generic application of the term “stress” masks the underlying reasons why certain studies identify significant associations while others do not [2,69]. In the present review, 60% (15 studies) of investigations relied exclusively on objective exposure via life-event counts, while only 16% (4 studies) utilized validated psychometric scales to capture the subjective response.

This “measurement mismatch” is evident when comparing classic and contemporary paradigms. Classic investigations into GD, such as the population-based works of Winsa et al. [17] and Radosavljević et al. [23], established a robust link between the volume of external negative events and disease onset. However, by focusing almost exclusively on exposure counts, these studies did not account for the depth of the individual's psychobiological reaction [2]. Conversely, more recent studies, such as those by Singh et al. [33] and Puttaswamy et al. [39], utilized the Perceived Stress Scale (PSS) to measure the degree to which patients felt “overwhelmed”. While this latter approach effectively captures the stress response, it often fails to inventory the objective stressors that triggered it, creating a measurement dimension that is not directly interchangeable with event inventories [2].

This gap offers an explanation for the negative results reported in large-scale prospective research. Longitudinal studies focusing solely on stressor exposure—such as the objective count of daily hassles or life events in healthy relatives of AITD patients—failed to demonstrate that these events trigger the initial de novo development of thyroid autoantibodies [18,19]. In the cohorts analyzed by Effraimidis et al. [18] and Strieder et al. [19], the quantification of event volume and general affect likely failed to capture the specific, high-intensity psychobiological response required to effectively dysregulate the HPT axis.

The comparative analysis by Vaivode et al. [31] exemplifies the appropriateness of assessing both stress dimensions to detect associations that might otherwise be overlooked. Their findings revealed no significant difference between AITD patients and healthy controls in the total number of stressful life events (exposure); however, patients exhibited a significantly higher negative stress burden (subjective response), which correlated directly with antibody titers and oxidative stress markers. This demonstrates that if only exposure had been evaluated, the study would have yielded negative results. Consequently, the findings of Vaivode et al. [31] suggest that the selection of stress assessment tools should be phenotype-informed. While objective life-event inventories may suffice to identify triggers in Graves' disease, the use of validated psychometric scales to capture the subjective stress response appears essential in Hashimoto's thyroiditis, where stress functions as an amplifier of disease activity.

### 4.3 Neuroendocrine mechanisms and biological embedding

The relationship between psychological stress and the HPT axis is characterized by acute reactivity rather than chronic baseline shifts [43]. The transient TSH surge observed in experimental settings suggests that thyroid function is dynamically responsive to situational overload [34,43].

Furthermore, our synthesis of early-life stress provides a distal framework for adult autoimmunity. Exposure to childhood adversity—particularly emotional neglect—may lead to the “biological embedding” of stress through chronic HPA-axis dysregulation and “wear and tear” on the immune system [2,69]. This supports a vulnerability model where early-life exposure shapes the adult subjective stress response, which, in the presence of specific genetic alleles such as HLA-Cw7, facilitates the breakdown of immunological tolerance [29,35].

### 4.4 An integrative phenotype-specific model of stress and thyroid autoimmunity

To integrate the consolidated evidence, we propose an integrative conceptual model in which the relationship between stress and AITD is understood as conditional on clinical phenotype rather than uniform across disease types (Fig. 2). Rather than a single continuous trajectory of physiological mobilization and exhaustion, the model depicts two qualitatively distinct pathways: a predominantly precipitating pathway for Graves' disease, and an amplification/feedback pathway for Hashimoto's thyroiditis.

**Fig. 2:**
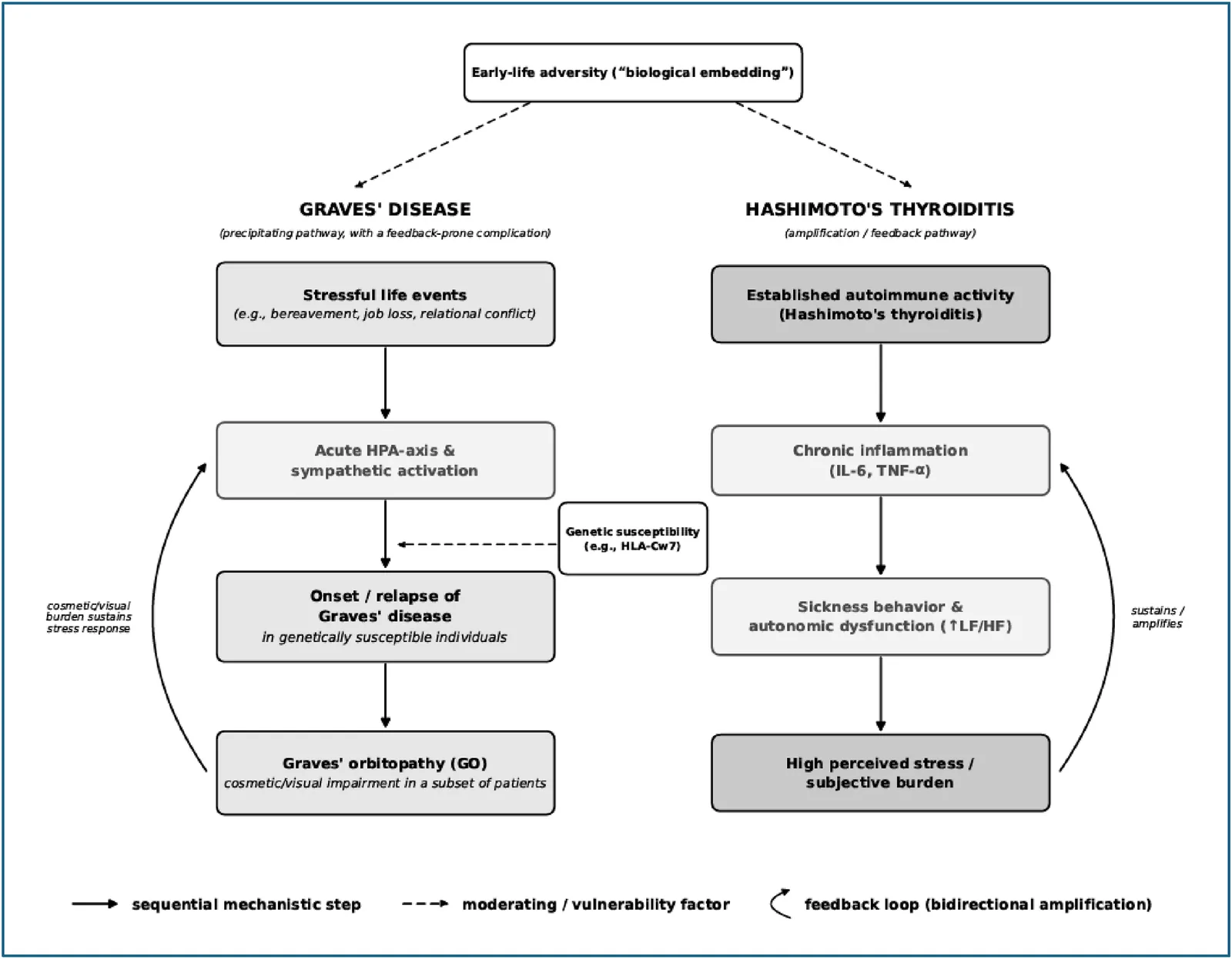
**Phenotype-specific stress pathways in autoimmune thyroid disease**. The diagram summarizes, for each clinical phenotype, the qualitative pattern of associations identified across the reviewed studies. In Graves' disease, stressful life events precede disease onset or relapse via acute HPA-axis and sympathetic activation, an effect moderated by genetic susceptibility; a subset of patients develops Graves' orbitopathy, whose cosmetic and visual impact can itself sustain or amplify the stress response. In Hashimoto's thyroiditis, established autoimmune activity sustains chronic inflammation and autonomic dysfunction, associated with a heightened subjective stress burden that may further reinforce disease activity. Early-life adversity is represented as an influence on both pathways through the “biological embedding” of stress reactivity. Arrows denote qualitative associations and mechanistic links reported in the reviewed literature.

Crucial to this framework is the methodological distinction between stressor exposure (objective events) and the subjective stress response (psychological burden). Consistent with established theoretical frameworks, the transition from adaptive resilience to allostatic overload is not determined by the volume of stressors alone, but is significantly mediated by the individual's subjective appraisal and the perceived lack of control over environmental demands [2,69,71]. Early-life stress is represented as a distal influence common to both pathways, resetting the individual's baseline biological regulation threshold through “biological embedding” of the immune transcriptome before adult stressors occur [69].

These patterns, as inferred from the reviewed evidence, suggest that GD is best characterized by an acute, alarm-like mobilization [72]: stressful life events precipitate acute HPA-axis and sympathetic activation, culminating in the onset or relapse of thyrotoxicosis in genetically susceptible individuals. While the majority of GD studies rely on case-control designs that limit causal inference, the prospective findings of Fukao et al. [14] regarding relapse and recent Mendelian randomization data [48] strengthen the hypothesis that high-intensity stressors act as clinical precipitants in susceptible individuals. Notably, a self-sustaining allostatic load [5] emerges only in the subset who develop Graves' orbitopathy, whose cosmetic and visual burden itself sustains or amplifies the stress response [30,45] — a feedback loop that this predominantly unidirectional pathway otherwise lacks. In HT, by contrast, this chronic allostatic load appears intrinsic to the phenotype itself: established autoimmune activity sustains chronic inflammation and autonomic dysfunction that is, in turn, associated with a heightened subjective stress burden capable of further reinforcing disease activity.

Information stemming from large-scale genetic and proxy-stress studies not included in the present review further substantiate this argument. They offer additional biological basis for the observed phenotype-dependent associations. For instance, Mendelian randomization analyses—a method that strengthens causal inference—have recently suggested a modest causal link between stress-related disorders (such as Post-Traumatic Stress Disorder - PTSD) and GD, while finding no evidence of a similar causal relationship with HT [48]. This causal link identified between PTSD and GD underscores the role of high-intensity stressor exposure as a potential trigger. Unlike the diffuse psychological burden often seen in HT, PTSD is fundamentally rooted in discrete traumatic events that mirror the ‘negative life events' (e.g., bereavement, workplace changes, or interpersonal conflicts) that historically characterize the onset of GD [17,23]. Furthermore, large-scale genomic data have identified a significant genetic overlap and positive correlation between stress-related disorders and thyroid autoimmunity, sharing biological pathways such as G-protein–coupled receptor signaling [49].

Within this conditional framework, genetic susceptibility would be a prerequisite for disease and also a dynamic moderator that dictates the individual's activation threshold. Evidence from HLA typing indicates that specific alleles, such as HLA-Cw7, function as markers of increased vulnerability, significantly influencing the biochemical severity and clinical trajectory of stress-triggered GD [28,35]. Carriers of the Cw7 allele exhibit higher serum FT3 levels at onset and are more prone to relapses after antithyroid drug withdrawal, suggesting that their genetic architecture may lower the activation threshold at which stressor exposure overwhelms immunological tolerance [35]. This genetic modulation is further evidenced by gene–environment interactions in GO, where specific polymorphisms synergize with environmental demands to trigger localized inflammation [44]. Therefore, the transition from adaptive resilience to allostatic overload is a personalized process, where the individual's genetic landscape determines the activation threshold at which the neuroendocrine–immune system shifts toward a maladaptive, autoimmune state [5,35,44].

### 4.5 Clinical implications: targeting the subjective stress response

Even though one should acknowledge that “psychological symptoms are not stress”, symptoms of mental distress (anxiety/depression) may serve as indicators of high subjective burden [2]. In GO, for instance, the negative impact of stressors is a major determinant of impaired quality of life and visual severity [30]. Therefore, psychosocial interventions—such as stress management or EMDR—should be viewed as strategies to modulate the stress response, potentially reducing the immunological amplification seen in HT and GD [16,38]. In addition, these strategies may be particularly relevant for individuals with markers of increased vulnerability, such as a history of early-life stress or genetic susceptibility, as a means to mitigate the “biological embedding” of adversity before it culminates in clinically relevant autoimmunity [29,32,35,42,69].

### 4.6 Limitations

Several limitations should be considered when interpreting this review. First, the high heterogeneity in study designs, populations, and thyroid outcomes limited comparability and precluded a meta-analysis. Most notably, a methodological conflation exists between exposure to stressful life events and the subjective stress response, with a disproportionate focus on life-event inventories (60% of studies) over validated psychometric scales to assess stress response (16%). This lack of interchangeable metrics likely masked phenotype-specific nuances in how stress modulates autoimmunity [2,69].

Second, the prevalence of cross-sectional and retrospective designs increases the risk of recall bias and limits causal inference, particularly regarding early-life adversity and event-triggered onset. Finally, the exclusion of non-English and non-peer-reviewed literature may have introduced language and publication bias, although the quality of the included evidence was mostly moderate to strong according to the EPHPP assessment.

### 4.7 Future directions

Future research should prioritize prospective longitudinal designs that adopt a dual-assessment approach, simultaneously measuring exposure to stressful life events and the subjective stress response to bridge the current ‘measurement gap’ [2]. Ideally, these studies should integrate validated psychometric scales with structured life-event inventories and repeated biological sampling of thyroid autoantibodies, inflammatory markers, and autonomic measures [7,8,69]. Accounting for key moderators—such as sex, age, and early-life adversity—will be essential to further clarify whether stress acts as a primary trigger for disease onset or an amplifier of clinical activity across different thyroid phenotypes [29,32].

## 5 Conclusion

The present systematic review demonstrates that the relationship between stress and autoimmune thyroid disease is distinctly phenotype-dependent and contingent on the stress construct evaluated. Consolidated evidence identifies stressful life events as a significant precipitating factor for Graves' disease onset and relapse. In contrast, Hashimoto's thyroiditis is more closely associated with a high subjective stress response, which acts as an amplifier of disease activity and autonomic dysfunction. These effects are modulated by early-life adversity and genetic susceptibility, which reset biological regulation thresholds through ‘biological embedding’. These findings underscore the clinical necessity of integrating psychosocial assessments into thyroid management, urging greater awareness among health care professionals of these dimensions as essential components of personalized patient care.

## CRediT authorship contribution statement

**José Manuel Comprido:** Writing – review & editing, Software, Investigation, Data curation. **Ana Carolina D. Salina:** Writing – review & editing, Software, Methodology, Investigation, Formal analysis, Conceptualization. **Vitor Vasconcellos F. da Silva:** Writing – review & editing, Methodology, Investigation, Conceptualization. **Paulo Roberto Haidamus de O. Bastos:** Writing – review & editing, Visualization, Validation. **Renata Boschi Portella:** Writing – review & editing, Visualization, Validation, Investigation, Formal analysis. **Danielle Bogo:** Writing – review & editing, Visualization, Validation. **Marco Aurélio Vinhosa Bastos:** Writing – review & editing, Writing – original draft, Supervision, Resources, Project administration, Methodology, Investigation, Formal analysis, Data curation, Conceptualization.

## Funding

The author declare that financial support was received for the research and/or publication of this article. This study was financed in part by the Universidade Federal de Mato Grosso do Sul – Brazil (UFMS), by the Coordenação de Aperfeiçoamento de Pessoal de Nível Superior – Brazil (CAPES) (Finance Code 001), and by the Fundação de Apoio ao Desenvolvimento do Ensino, Ciência e Tecnologia do Estado de Mato Grosso do Sul (Fundect).

## Declaration of competing interest

The authors declare that they have no known competing financial interests or personal relationships that could have appeared to influence the work reported in this paper.
