# Supplementary material for: Psychosocial stress and autoimmune thyroid disease: a systematic review of clinical phenotypes and vulnerability factors
Source: Compr Psychoneuroendocrinol. 2026 Sep 22;28:100382. doi: 10.1016/j.cpnec.2026.100382 (PMC13676715; doi:10.1016/j.cpnec.2026.100382)
Supplement: Supplementary file 1 [file mmc1.docx]

**The association between stress and autoimmune thyroid disease: a systematic review**

**(Supplementary Material)**

Table S1 – Detailed search strategies in each database.

| Database | Search strategy | Results |
| --- | --- | --- |
| PubMed | (stress[MeSH Terms] OR psychological stress[Title/Abstract] OR psychosocial stress[Title/Abstract] OR chronic stress[Title/Abstract] OR perceived stress[Title/Abstract] OR stressful life events[Title/Abstract]) AND (autoimmune thyroid disease[Title/Abstract] OR Hashimoto thyroiditis[MeSH Terms] OR Hashimoto disease[Title/Abstract] OR Graves disease[MeSH Terms]). | 36 |
| PsycINFO | (stress OR psychological stress OR psychosocial stress OR chronic stress OR perceived stress OR life stress OR stressful life events) AND (autoimmune thyroid disease OR Hashimoto thyroiditis OR Hashimoto disease OR Graves disease) (Search restricted to: Abstract). | 36 |
| Scopus | (stress OR psychological stress OR psychosocial stress OR chronic stress OR perceived stress OR stressful events OR stress exposure) AND (Hashimoto thyroiditis OR Hashimoto disease OR Graves disease OR autoimmune thyroid) (Limit to: Title, Abstract, Keywords). | 29 |
| Embase | ('psychological stress':ti,ab,kw OR 'psychosocial stress':ti,ab,kw OR 'chronic stress':ti,ab,kw OR 'perceived stress':ti,ab,kw OR 'stressful life events':ti,ab,kw) AND ('autoimmune thyroid disease':ti,ab,kw OR 'hashimoto thyroiditis':ti,ab,kw OR 'hashimoto disease':ti,ab,kw OR 'graves disease':ti,ab,kw). | 49 |

Table S2 - Quality assessment (EPHPP) of studies examining the association between stress and hormones of the hypothalamic–pituitary–thyroid axis.

|  | A  (Selection Bias) | B  (Study Design) | C  (Con - founders) | D  (Blinding) | E  (Data Collection Method) | F  (With - drawals/ Dropouts) | Global Rating |
| --- | --- | --- | --- | --- | --- | --- | --- |
| (Fischer et al., 2019) | 3 | 1 | 1 | 3 | 1 | 1 | 3 |
| (Hong & Lee, 2022) | 2 | 2 | 1 | NA | 1 | NA | 1 |
| (Puttaswamy et al., 2024) | 1 | 3 | 2 | NA | 1 | NA | 2 |

1-Strong; 2-Moderate; 3-Weak; NA-Non-applicable

Table S3 - Quality assessment (EPHPP) of studies examining the association between stress and autoimmune thyroid disease.

|  | A  (Selection Bias) | B  (Study Design) | C  (Con - founders) | D  (Blinding) | E  (Data Collection Method) | F  (With - drawals/ Dropouts) | Global Rating |
| --- | --- | --- | --- | --- | --- | --- | --- |
| (Strieder et al., 2005) | 2 | 3 | 2 | NA | 1 | NA | 2 |
| (Effraimidis et al., 2012) | 2 | 2 | 2 | NA | 1 | NA | 1 |
| (Vaivode et al., 2023) | 2 | 3 | 1 | NA | 1 | NA | 2 |

1-Strong; 2-Moderate; 3-Weak; NA-Non-applicable

Table S4 - Quality assessment (EPHPP) of studies examining the association between stress and Graves’ disease.

|  |  | A  (Selection Bias) | B  (Study Design) | C  (Con - founders) | D  (Blinding) | E  (Data Collection Method) | F  (With - drawals/ Dropouts) | Global Rating |  |
| --- | --- | --- | --- | --- | --- | --- | --- | --- | --- |
|  | (Stewart et al., 1985) | 2 | 2 | 3 | NA | 3 | NA | 3 |  |
|  | Winsa et al.,1991 (Sweden) | 2 | 2 | * | NA | * | NA | * |  |
|  | Kung, 1995 (Hong Kong) | 2 | 2 | 2 | NA | 1 | NA | 1 |  |
|  | (Radosavljević et al., 1996) | 2 | 2 | 2 | NA | 1 | NA | 1 |  |
|  | (Yoshiuchi et al., 1998) | 2 | 2 | * | NA | * | NA | * |  |
|  | (Matos Santos et al., 2001) | 2 | 2 | 3 | NA | 1 | NA | 2 |  |
|  | (Matos Santos et al., 2002) | 2 | 2 | 2 | NA | 1 | NA | 1 |  |
|  | (Fukao et al., 2003) | 2 | 2 | 2 | NA | 1 | NA | 1 |  |
|  | (Vita et al., 2015) | 2 | 2 | 2 | NA | 1 | NA | 1 |  |
| 1-Strong; 2-Moderate; 3-Weak; NA-Non-applicable | | | | | | | | | |

* Assessment impossible due to only the abstract being available.

Table S5 - Quality assessment (EPHPP) of studies examining the association between stress and Graves’ ophtalmopathy.

|  |  | A  (Selection Bias) | B  (Study Design) | C  (Con - founders) | D  (Blinding) | E  (Data Collection Method) | F  (With - drawals/ Dropouts) | Global Rating |  |
| --- | --- | --- | --- | --- | --- | --- | --- | --- | --- |
|  | (Kahaly et al., 2005) | 2 | 2 | 1 | NA | 1 | NA | 1 |  |
|  | (Zhou et al., 2017) | 2 | 2 | 2 | NA | 3 | NA | 2 |  |
| 1-Strong; 2-Moderate; 3-Weak; NA-Non-applicable | | | | | | | | | |

Table S6 - Quality assessment (EPHPP) of studies examining the association between stress and Hashimoto’s thyroiditis.

|  |  | A  (Selection Bias) | B  (Study Design) | C  (Con - founders) | D  (Blinding) | E  (Data Collection Method) | F  (With - drawals/ Dropouts) | Global Rating |  |
| --- | --- | --- | --- | --- | --- | --- | --- | --- | --- |
|  | (Markomanolaki et al., 2019) | 2 | 1 | 2 | 3 | 1 | 1 | 2 |  |
|  | (Singh et al., 2024) | 2 | 2 | 2 | NA | 1 | NA | 1 |  |
|  | (Macarenco et al., 2026) | 2 | 1 | 1 | 2 | 1 | 1 | 1 |  |
| 1-Strong; 2-Moderate; 3-Weak; NA-Non-applicable | | | | | | | | | |

Table S7 - Quality assessment (EPHPP) of studies examining the association between early life stress and autoimmune thyroid disease.

|  |  | A  (Selection Bias) | B  (Study Design) | C  (Con - founders) | D  (Blinding) | E  (Data Collection Method) | F  (With - drawals/ Dropouts) | Global Rating |  |
| --- | --- | --- | --- | --- | --- | --- | --- | --- | --- |
|  | (Corso et al., 2023) | 2 | 2 | 2 | NA | 1 | NA | 1 |  |
|  | (Aydogan et al., 2024) | 2 | 2 | 2 | NA | 1 | NA | 1 |  |
|  | (Köhler-Forsber et al., 2025) | 2 | 2 | 1 | NA | 1 | NA | 1 |  |
| 1-Strong; 2-Moderate; 3-Weak; NA-Non-applicable | | | | | | | | | |

Table S8 - Quality assessment (EPHPP) of studies examining the association between stress, autoimmune thyroid disease, and genetic markers.

|  |  | A  (Selection Bias) | B  (Study Design) | C  (Con - founders) | D  (Blinding) | E  (Data Collection Method) | F  (With - drawals/ Dropouts) | Global Rating |  |
| --- | --- | --- | --- | --- | --- | --- | --- | --- | --- |
|  | (Vita et al., 2017) | 3 | 2 | 2 | NA | 2 | NA | 2 |  |
|  | (Yang et al., 2018) | 2 | 2 | 1 | NA | 3 | NA | 2 |  |
| 1-Strong; 2-Moderate; 3-Weak; NA-Non-applicable | | | | | | | | | |

Table S9. Details of studies examining the association between stress and hormones of the hypothalamic–pituitary–thyroid axis

| **Authors/ Location** | **Characteristics of the samples (n)** | **Sex (M/F)**  **Age range (years)** | **Study design** | **Exposure /Intervention** | **Outcomes** | **Main findings** |
| --- | --- | --- | --- | --- | --- | --- |
| Fischer et al., 2019  (Austria, Germany, Switzerland) | Healthy individuals: 30 | All female    37 ± 13 y | Randomized, controlled cross-over intervention study | Intervention: Trier Social Stress Test (TSST)  vs.  Non-stressful control task  *Note: single-arm cross-over design | Total T3, total T4 and TSH, with blood sampling at four time points, and psychological stress assessed using a visual analogue scale (VAS). | Thyroid-stimulating hormone (TSH) concentrations rise significantly in response to the TSST, with a peak 20 min after stressor onset, and a steady decline thereafter. No such response in the control session. TSST did not raise T3 or T4. |
| Hong & Lee, 2022  (South Korea) | Post-thyroidectomy patients: 106  Note:  *Total thyroidectomy: 58  *Partial thyroidectomy: 48 | (22/ 84)  46.7 ± 10 y | Prospective cohort study (single-center, ~22 months of follow-up) | Exposure:  Stress exposure was assessed using multiple instruments administered twice, including daily stress (KDSI*), major life events (SRRS*), and recent perceived stress (SOSS*), complemented by an open-ended questionnaire. Questionnaires were applied at a mean of 51 months post-thyroidectomy and repeated after approximately 6.8 months. | Changes in TSH, free T4 and T3 levels. Anti-thyroid antibodies not assessed. | TSH levels were significantly associated with stress, particularly when stress was assessed using a categorical version of the SOSS complemented by an open-ended questionnaire. Higher stress categories were associated with increased TSH levels (P < 0.05), whereas continuous stress measures showed weak or no correlations. TSH was inversely associated with the extent of thyroidectomy. Authors argue that, in post-thyroidectomy patients, TSH may reflect hypothalamic–pituitary axis responsiveness to stress. |
| Puttaswamy et al., 2024  (India) | Healthy individuals with no family history of autoimmune thyroid disease (ATD): 114  Two subgroups:  *Normal thyroid function: 93  *Subclinical hypothyroidism (SCH): 21 | All female  34.2 ± 6.1 y | Cross-sectional | Exposure:  Perceived stress levels, measured using the Perceived Stress Scale (PSS). | Total T3, total T4, TSH, anthropometric and vital measures (age, BMI, pulse rate, blood pressure), and sociodemographic and lifestyle factors. | No significant correlation was found between perceived stress (PSS) and TSH levels. Women with subclinical hypothyroidism were older, had higher BMI, and were more likely to be married. Overall stress levels did not differ between groups, although low stress scores were more frequent among women with normal thyroid function (19.4% vs. 9.5%; p = 0.025). |

* KDSI: Korean version of the Daily Stress Inventory / SRRS: Social Readjustment Rating Scale/ SOSS: Stress Overload Scale-Short form /

Table S10. Details of studies examining the association between early life stress and autoimmune thyroid disease.

| **Authors/ Location** | **Characteristics of the samples (n)** | **Sex (M/F)**  **Age range (years)** | **Study design** | **Exposure /Intervention** | **Outcomes** | **Main findings** |
| --- | --- | --- | --- | --- | --- | --- |
| Corso et al., 2023  (Switzerland) | Autoimmune thyroid disease (ATD): 78  Healthy controls: 130 | All female  53 ± 13 y | Case-control | Exposure:  Presence or absence of ATD. | The frequencies or prevalences of different categories of early life stress, as assessed by the Childhood Trauma Questionnaire (CTQ). This instrument assesses five domains: emotional neglect, physical neglect, emotional abuse, physical abuse, and sexual abuse. | Women with autoimmune thyroid disease showed higher frequencies of emotional neglect and emotional abuse compared to controls, although these differences were of borderline statistical significance. No significant differences were observed for other forms of childhood trauma (sexual abuse, physical abuse, or physical neglect). |
| Aydogan et al., 2024  (Turkey) | Hashimoto thyroiditis (HT): 52  Healthy controls: 31 | HT group: (11/ 41)  39.5 ± 13 y  Control group:  (11/ 20)  34.7 ± 12 y | Case-control | Exposure:  Presence of HT as assessed by biochemical markers (anti-TPO, TSH, fT3, fT4)  and thyroid ultrasonography. | Retrospective assessment of childhood trauma using the Turkish version of the Childhood Trauma Questionnaire (CTQ), including emotional, physical, and sexual abuse, as well as emotional and physical neglect. | Emotional neglect scores were significantly higher in HT patients compared to controls and were positively correlated with anti-TPO levels (r = 0.26; p = 0.02). No significant differences were found for other childhood trauma subtypes, and no associations were observed with thyroid function tests. Authors suggest that emotional neglect may contribute to the etiology of HT. |
| Köhler-Forsberg et al., 2025  (Denmark, Iceland, Sweden, China, and USA) | The study utilized data from two cohorts: the Icelandic Stress-And-Gene-Analysis (SAGA) cohort and the UK Biobank (UKB).  SAGA cohort: 22,423 individuals  UK Biobank: 86,492individuals | All female  SAGA cohort:  43.8 ± 13 y  UK Biobank 55. ± 7.6 y | Observational study utilizing both cross-sectional and prospective data  (mean duration of follow-up was 4.2 y) | Adverse childhood experiences (ACEs), self-reported, including emotional neglect, physical neglect, emotional abuse, physical abuse, and sexual abuse. The primary exposure variables were the total ACE score (0–5) and individual ACE categories. | Included prevalence and incidence of 12 autoimmune diseases (e.g., thyroid disease, type 1 diabetes, multiple sclerosis). Mediators included self-reported symptoms of depression, anxiety, and post-traumatic stress disorder (PTSD). | ACEs were associated with increased prevalence of autoimmune diseases in a dose-response manner (prevalence ratio = 1.10 per ACE), with strong associations observed for several conditions, including thyroid disease. Specific ACEs (sexual abuse, physical and emotional neglect) were consistently linked to higher risk. Approximately 28% of this association was mediated by depression, anxiety, and PTSD symptoms, with depression as the main mediator. |

Table S11. Details of studies examining the association between stress, autoimmune thyroid disease, and genetic markers.

| **Authors/ Location** | **Characteristics of the samples (n)** | **Sex (M/F)**  **Age range (years)** | **Study design** | **Exposure /Intervention** | **Outcomes** | **Main findings** |
| --- | --- | --- | --- | --- | --- | --- |
| Vita et al., 2017  (Italy) | Caucasian patients with stress-related Graves’ disease (GD): 58  Caucasian euthyroid healthy controls: 130  Note: All 58 patients experienced at least one stressful event (psychological, infectious, or physical) that preceded the onset or recurrences of hyperthyroidism within 12 months. | GD group:  (22/ 36)  35.4 ± 14.6 y (at onset of hyperthyroidism)    Control group:  (65/ 65)  Age-matched | Observational study utilizing both cross-sectional and prospective data  (patients were followed up for at least 5 y after antithyroid drug withdrawal (median = 13 y) | Exposure:  *Cross-sectional component: Presence of GD  *Prospective component: Recurrence of hyperthyroidism during treatment (exacerbation) or after antithyroid drug withdrawal (relapse). | HLA associations with GD susceptibility, clinical course (remission/exacerbation/relapse), age at onset, and biochemical severity. | HLA alleles B8, DR3, DR4, DQ2 were more frequent in GD patients, while B14 appeared protective. Certain alleles were associated with disease course: Cw7/C07 was linked to exacerbation/relapse, whereas Cw6/C06 was more frequent in remission, suggesting a protective effect. Some alleles also influenced age at onset (earlier in B35 and Cw7 carriers, later in DR1 carriers) and biochemical severity (higher FT3 in Cw7 carriers). HLA profile may help predict disease course after antithyroid drug withdrawal. |
| Yang et al., 2018  (China) | Graves' disease (GD) with orbitopathy (GO): 305  GD patients without orbitopathy: 283 | GO group:  (122/ 183)  Age < 30: 76%,  Age ≥ 30: 24%  No GO group:  (123/ 160)  Age < 30: 91% Age ≥ 30: 9% | Case-control | Exposure:  Presence of GO | Genetic polymorphisms in the LFA-1/ICAM-1/GSK-3β pathway and clinical/environmental factors (age, BMI, smoking, family history of thyroid disease, stressful work environment, and I¹³¹ therapy). | Significant gene–environment interactions were observed in GO susceptibility. A stressful work environment was associated with increased risk through interaction with specific SNP combinations (rs6438552, rs12716977, and rs2230433). |
